# 2193: Targeting MELK in acute lymphoblastic leukemia, new therapeutic approach

**DOI:** 10.1017/cts.2017.209

**Published:** 2018-05-10

**Authors:** Houda Alachkar, Martin Mutonga, Amanda de Albuquerque, Rucha Deo, Gregory Malnassy, Yusuke Nakamura, Wendy Stock

## Abstract

OBJECTIVES/SPECIFIC AIMS: Unlike the high cure rates (90%) of children with acute lymphoblastic leukemia (ALL), that of adults is still lagging behind and better therapies are needed. Maternal embryonic leucine-zipper kinase (MELK) is aberrantly upregulated in cancer, and implicated in cancer stem cell survival. A recent study has identified FOXM1, a MELK substrate, as a therapeutic target in B cell ALL (B-ALL). Thus, we hypothesized that MELK may act as a therapeutic target in ALL via targeting FOXM1 activity. METHODS/STUDY POPULATION: Western blot and qPCR were used to assess MELK expression in 14 ALL cell lines. Knock-down and kinase inhibition approaches targeting MELK expression and function, followed by CCK-8 and Annexin V (flow cytometry) assays to measure cell viability and apoptosis, respectively. RESULTS/ANTICIPATED RESULTS: MELK was significantly upregulated in patients with ALL (oncomine data analysis). MELK was also significantly higher in B-ALL and T-ALL cell lines compared with that in blood cells of healthy donors. MELK knock-down significantly decreased cell viability (40%–70%, *p*<0.05, Fig. 1) in ALL cells, and induced apoptosis (~40%). OTS167, a potent MELK inhibitor exhibited cytotoxic activities in both B and T-ALL cells. The IC50 of OTS167 ranged from 20 to 60 nM; we also found a significant increase in apoptosis (*p*<0.05). Mechanistically, MELK inhibition resulted in decrease of FOXM1 protein levels 3 hours post-treatment. DISCUSSION/SIGNIFICANCE OF IMPACT: MELK is highly expressed in ALL and represents a novel therapeutic target likely via modulating FOXM1 activity. Functional and mechanistic studies will complement and ensure the success of the undergoing Phase I/II clinical trial of OTS167 in patients with refractory or relapsed AML, ALL, and other advanced hematologic malignancies.

